# Cardiovascular response to postural perturbations of different intensities in healthy young adults

**DOI:** 10.14814/phy2.15299

**Published:** 2022-05-09

**Authors:** Patrick Siedlecki, J. Kevin Shoemaker, Tanya D. Ivanova, S. Jayne Garland

**Affiliations:** ^1^ 6221 School of Kinesiology Western University London Ontario Canada; ^2^ 6221 Faculty of Health Sciences Western University London Ontario Canada; ^3^ 6221 Department of Physiology & Pharmacology Western University London Ontario Canada

**Keywords:** central command, autonomic nervous system, postural control, baroreflex

## Abstract

The ability to regain control of balance is vital in limiting falls and injuries. Little is known regarding how the autonomic nervous system responds during recovery from balance perturbations of different intensities. The purpose of this study was to examine the cardiovascular response following a standing balance perturbation of varying intensities, quantify cardiac baroreflex sensitivity (cBRS) during standing perturbations, and to establish the stability of the cardiac baroreflex during quiet standing before and after balance disturbances. Twenty healthy participants experienced three different perturbation intensity conditions that each included 25 brief posteriorly‐directed perturbations, 8–10 s apart. Three perturbation intensity conditions (low, medium, high) were given in random order. Physiological data were collected in quiet stance for 5 min before testing (Baseline) and again after the perturbation conditions (Recovery) to examine baroreflex stability. Beat‐to‐beat heart rate (HR) and systolic blood pressure (SBP) analysis post‐perturbation indicated an immediate acceleration of the HR for 1–2 s, with elevated SBP 4–5 s post‐perturbation. Heart rate changes were greatest in the medium (*p* = 0.035) and high (*p* = 0.012) intensities compared to low, while there were no intensity‐dependent changes in SBP. The cBRS was not intensity‐dependent (*p* = 0.402) but when perturbation conditions were combined, cBRS was elevated compared to Baseline (*p* = 0.046). The stability of baseline cBRS was excellent (ICC = 0.896) between quiet standing conditions. In summary, HR, but not SBP or cBRS were intensity‐specific during postural perturbations. This was the first study to examine cardiovascular response and cBRS to postural perturbations.

## Graphical abstract

We examined the cardiovascular response following a standing balance perturbation of varying intensities, and quantified cardiac baroreflex sensitivity during standing perturbations. Beat‐to‐beat heart rate and systolic blood pressure analysis found an immediate acceleration of the heart rate, with elevated blood pressure lagging. The cardiac baroreflex sensitivity was not intensity‐dependent.

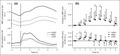

**New Findings**

**What is the central question of this study?**

- What is the cardiovascular response to postural perturbations and is it intensity‐dependent?

**What is the main finding and its importance?**

- We observed a hemodynamic response and recovery after the onset of a postural perturbation (SBP and HR) that was intensity‐dependent (HR). Increased HR immediately post‐perturbation was followed by an increase in blood pressure and subsequent decrease in HR 4–5 s post‐perturbation suggesting baroreflex‐mediated influence on HR. The baroreflex may serve as a compensatory mechanism to assist upright stability.

## 1 INTRODUCTION

It is reported that 37.3 million falls occur each year that require medical attention and that the risk of sustaining a fall in adults increases with age (World Health Organization, 2018). Standing balance relies on the central nervous system's control of skeletal muscle torques to regulate the center of mass following an external perturbation (Ogaya et al., 2016; Silva et al., 2018; Winter et al., 1998). Additionally, the autonomic nervous system modulates cardiovascular function during postural adjustments (Carpenter et al., 2006; Olufsen et al., 2005). Recent findings illustrated temporal associations between systolic blood pressure (SBP), muscle activity, and postural control during quiet standing (Garg et al., 2014), supporting the concept of coupling between cardiovascular and skeletal muscle control to support balance. To date, the role of the baroreflex in supporting postural blood pressure adjustments are limited to quiet standing conditions. The cardiovascular adjustments to more severe postural perturbations, and how they relate to baroreflex function are not known.

Automatic adjustments to physical stress can be centrally‐ and/or reflex‐mediated (Dombrowski et al., 2018). The role of baroreflex regulation of blood pressure is complex, representing contributions from central neural systems (McCloskey & Mitchell, 1972), as well as ascending feedback from baroreceptor, chemoreceptor, and mechanosensors located in skeletal muscle (see Raven et al., 2019). The gain or sensitivity of the reflex is highly modifiable and can change rapidly due to its relationship to cardiovagal dominance of heart rate (HR; La Rovere et al., 2001). This process of baroreflex engagement represents a critical element of achieving rapid cardiovascular adjustments to exercise (Raven et al., 2019; Zamir et al., 2017) and postural shifts (Schwartz & Stewart, 2012). Whether changes in cardiac baroreflex sensitivity (cBRS) accompany adjustments to acute postural perturbations is not known. Therefore, HR and blood pressure responses could be explained by baroreflex involvement in hemodynamic regulation following postural perturbations.

The primary purpose of this study was to examine the cardiovascular response following a standing balance perturbation of varying intensities. Secondary aims were to quantify cardiac baroreflex sensitivity during standing perturbations and to establish the stability of the baroreflex during quiet standing before and after balance disturbances. The study tested the hypothesis that postural perturbations would induce intensity‐dependent increases in heart rate and blood pressure that would coincide with an elevated cardiac baroreflex sensitivity.

## 2 METHODS

### 2.1 Ethical approval

Twenty young adults, who reported no neurological disorders, respiratory diseases, or musculoskeletal disorders, and considered themselves to be healthy completed the study. Although medication use and the presence of cardiovascular disease were not used as exclusion criteria, all participants were normotensive, had normal resting HR while standing, and had a body mass index under 30 kg/m^2^. Participants were asked to fast for a minimum of 4 h, to refrain from consuming caffeinated and alcoholic beverages, and to avoid any strenuous physical activity for 24 h prior to their scheduled appointment. Participants provided written consent to the study procedures that had been approved by the University of Western Ontario Health Sciences’ Research Ethics Board (#110471). The study conformed to the standards set by the Declaration of Helsinki, except for registration in a database.

### 2.2 Experimental protocol

Participants began by filling out the Self‐Evaluation Breathing Questionnaire (SEBQ‐2) to identify respiratory‐related symptoms that could be associated with impaired breathing (Courtney & van Dixhoorn, 2014). The 25‐item self‐report questionnaire ranks statements regarding symptoms on a 0–3 Likert scale, 0 indicating the statement is not true and 3 indicating the statement is true and that the symptoms occur very frequently. Overall scores greater than 11 out of 75 indicate the possibility of respiratory‐related problems (Courtney & van Dixhoorn, 2014). The Community Balance and Mobility Scale (CB&M) was performed to assess ambulatory balance (e.g., unilateral stance, tandem walking, hopping, walking forwards and backwards, etc.). Each of the 19 items (including one bonus point) were rated by the same researcher (P.S.) on a scale of 0 to 5, with 96 as the best overall score (Howe et al., 2006).

Participants completed the perturbation testing using the Gait in Real‐time Analysis Interactive Lab (GRAIL; Motekforce Link, Amsterdam, Netherlands) system. The GRAIL consists of a split‐belt treadmill and a 180‐degree virtual reality screen in a quiet, dimly lit room. The virtual reality screen, positioned in front of the participant, displayed a cobblestone path through an open grass field. The movement of the image was linked to the treadmill belts (e.g., if the belts moved posteriorly, the path on the screen would appear to be moving towards the participant). The virtual reality screen was used to create a more realistic experimental environment compared to traditional laboratory settings (Teel et al., 2016; Teel & Slobounov, 2015). Participants wore an upper‐body safety harness that did not provide any body weight support but would prevent a fall. Participants stood on the treadmill and were fitted with a 3‐Lead Bio Amp ECG (ADInstruments, Bella Vista, Australia), and a finger cuff with brachial Finometer sphygmomanometer (Finapres Medical System, Amsterdam, The Netherlands) placed on the right arm to collect cardiovascular measures throughout the experiment. An arm sling was worn by participants to restrict movement of the right arm as the sphygmomanometer was sensitive to movement.

Postural perturbations were introduced using simultaneous posteriorly‐directed movements of both treadmill belts with a 300 ms duration, which caused a forward movement of the participant's center of mass. Participants were instructed to regain balance without taking a step or grasping the treadmill handles. If participants had to take a step, they were told to do so with their left leg. Participants were instructed to limit head movement by focusing on a fixation point (horizon) located on the virtual reality screen approximately 2.5 m in front of them. During a familiarization period, the perturbation velocity was increased in a stepwise fashion. Three perturbation intensity levels, low (LOW), medium (MED), and high (HIGH) were determined for each participant. The maximum velocity at which a participant was able to maintain balance without taking a step was selected as the MED condition. The LOW and HIGH conditions were determined to be 50% below and 50% above the MED velocity, respectively. Therefore, only the HIGH condition required a step to regain balance. There were no trials in which participants took a step in the LOW or MED conditions. The average treadmill belt velocities for the perturbation intensities were; 0.19 m/s for LOW, 0.35 m/s for MED, and 0.62 m/s for HIGH conditions with peak velocities being 0.31, 0.61, and 0.92 m/s for LOW, MED, and HIGH, respectively. The condition order was randomized, and each condition consisted of 25 perturbations of the same intensity level, delivered 8–10 s apart with a 1‐min rest between conditions. Each condition lasted 4–4.5 min. Participants began each condition standing in the middle of the treadmill track. Participants re‐positioned themselves only in the MED condition to avoid falling off the treadmill track every 5–10 perturbations. These corrections were made after the ~8 s period of cardiovascular reaction following the perturbation. The subsequent perturbation was delayed if re‐positioning occurred too close to the next planned perturbation.

The treadmill perturbations were triggered using an application created in the GRAIL software D‐flow (Motekforce Link, Amsterdam, Netherlands). The speed of each treadmill belt was recorded through a Phidget Analog 4‐output #1002_0B (Phidgets, Inc., Calgary, AB, Canada).

### 2.3 Blood pressure and heart rate response calculations

The blood pressure and electrocardiogram tracings, sampled at 1000 Hz (Powerlab 8/35; ADInstruments, Bella Vista, Australia) were used to derive the beat‐to‐beat SBP and HR, respectively and exported for further analysis. Belt velocity signals together with SBP and HR for each perturbation condition were imported into Spike2 v.8.13 (Cambridge Electronic Design Limited, Milton, England). The onset of each perturbation was determined by threshold‐crossing on the filtered left treadmill belt speed signals. The threshold was calculated as the point the signal reached two standard deviations (SD) above the mean in a 500 ms epoch prior to the perturbation. The beat‐to‐beat data were down‐sampled to 50 Hz and a 12 s window starting 4 s prior to the onset of the perturbation was selected for each trial. The SBP and HR data were normalized to each participant's SBP and HR at Baseline during quiet stance, respectively. Trials with artifacts in the SBP or HR tracings were excluded. Individual trials were averaged for each participant within each perturbation condition. Data from five minutes of quiet standing before and following the test trials were used for Baseline and Recovery periods, respectively.

### 2.4 Baroreflex sensitivity calculations

**FIGURE 1:**
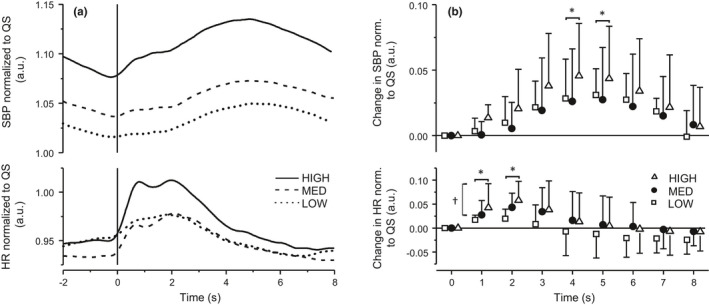
(a) Average beat‐to‐beat systolic blood pressure (SBP; top) and heart rate (HR; bottom) response for 2 s prior to the perturbation and 8 s post‐perturbation in the LOW (dotted line), MED (dashed line), and HIGH (solid line) intensity conditions. The vertical line represents the onset of the perturbation. Data are normalized to quiet stance. (b) Average change, normalized to quiet stance, in beat‐to‐beat SBP (top) and HR (bottom) for each second post‐perturbation in LOW (open square), MED (filled circle), and HIGH (open triangle) intensity conditions. *Significant differences from time 0 (*p* < 0.05), ^†^LOW was significantly depressed compared to MED and HIGH (*p* < 0.05)

One of our aims was to determine changes in cBRS during the period immediately following the balance perturbation until re‐stabilization. All calculations were performed with MATLAB R2019b (MathWorks Inc., Natick, MA, USA). For baseline conditions, the sequence method (Parati et al., 2000) was applied to the beat‐to‐beat R‐R interval and SBP data from Baseline (5 min) and Recovery (5 min) periods. Following observations of the rapid but transient HR and SBP changes after the perturbations (see Figure 1a), the sequence method was applied to a time period (1 to 8 s) following the onset of each perturbation (+0.4 s if the onset occurred between 2 heart beats). This method was adapted from Gabbett et al. (2001) to view the immediate cardiovascular response. Artifacts in R‐R interval and SBP, identified by visual inspection, occurred rarely, and only affected a single data point when present. These data points were replaced with interpolated data one cardiac cycle before and after the missing data point.

The sequence method calculated cBRS with a lag set at 0 beats using non‐normalized R‐R interval and SBP data. This lag was determined post hoc based on the average R‐R interval in each condition being <775 ms (Blaber et al., 1995). A sequence was determined as three or more consecutive cardiac cycles where R‐R interval and SBP increased or decreased together. A minimum change in R‐R interval (4 ms) and SBP (1 mmHg) between beats must have occurred to have been considered part of a sequence. The slopes of the regression line between R‐R interval and SBP were calculated for each sequence, and only sequences that had regression lines with *r* ^2^ > 0.85 were used. Cardiac baroreflex sensitivity was determined to be the average of the slopes of all sequences within the selected time periods (Bertinieri et al., 1985).

### 2.5 Statistical analysis

All statistical analyses were performed with SPSS v.25 (IBM SPSS, Armonk, NY, USA). To compare beat‐to‐beat SBP and HR response between perturbation conditions, the change between the SBP and HR values at each second after the perturbation and the perturbation onset was calculated. Separate two‐way repeated measures ANOVAs with condition (LOW, MED, HIGH) and time (0, 1…8 s) were performed to compare the change in SBP and HR for 8 s after a perturbation. One‐way repeated measures ANOVAs were used to determine the effect of perturbation intensity on cBRS. As no statistically significant differences in cBRS between perturbation intensities were found, the data were averaged across perturbation intensity. This also increased the number of sequences in the perturbation tasks (~53 sequences) so that they were closer to the total number of sequences found during Baseline (75 sequences). Paired samples *t*‐tests were used to determine the effect of perturbations (combined across intensity conditions vs. Baseline) on cBRS characteristics. Post‐hoc pairwise comparisons with Bonferroni corrections were performed following all ANOVAs. Paired samples *t*‐tests were used to examine the differences in cBRS between Baseline and Recovery periods. A mixed model, intraclass correlation coefficient (ICC) was used to determine the stability of the cardiac baroreflex between Baseline and Recovery quiet stance periods. The level of statistical significance was set to *p* = 0.05 for all analyses.

## 3 RESULTS

The participant characteristics are summarized in Table 1 and raw SBP and HR values for 4 s (~5 cardiac beats) pre‐perturbation and the maximum SBP and HR value in the 8 s after the onset of the perturbation can be found in Table 2.

**TABLE 1:** Participant characteristics

Age (years)	Sex^a^ (m/f)	Height (cm)	Weight (kg)	SEBQ scores (/75)	CB&M score (/96)
24.2 (3.3)	10/10	170.1 (8.9)	70.1 (12.6)	7.6 (6.1)	95.4 (1.0)

Note

Data presented as mean (SD).

Abbreviations: CB&M, community balance & mobility scale; SEBQ, aelf‐evaluation breathing questionnaire.

^a^ Number of participants

**TABLE 2:** Raw cardiovascular measures during perturbation tasks

	Low	Med	High
HR (bpm)			
Pre perturbation	82 (13)	81 (12)	82 (12)
Post perturbation peak	89 (13)	89 (13)	92 (12)
SBP (mmHg)			
Pre perturbation	122 (12)	124 (11)	129 (10)
Post perturbation peak	129 (12)	132 (13)	140 (12)

Note

Heart rate (HR; *n* = 18) and systolic blood pressure (SBP; *n* = 19) data presented as mean (SD) in LOW, MED, and HIGH conditions.

### 3.1 Perturbation intensity effect on cardiovascular response

Figure 1 presents the average SBP (*n* = 18) and HR (*n* = 19) responses to the three perturbation intensities (Figure 1a) together with the average change from perturbation onset (time 0) for each second post‐perturbation (Figure 1b). The SBP and HR data from one participant were excluded due to missing treadmill data and the SBP data were excluded from another participant in a single intensity condition due to movement artifacts caused by finger contractions occurring throughout the entire condition. There was a significant effect of *time* for SBP changes (*p* = 0.002). No interaction effect between *condition* and *time* (*p* = 0.156) or main effect of *condition* (*p* = 0.128) were found for SBP. Pairwise comparisons across conditions showed that SBP was elevated at the 4th and 5th s after the perturbation (*p* = 0.037 and *p* = 0.027, respectively), returning to baseline at 6 s. There were significant main effects of *time* (*p* = 0.001) and *condition* (*p* = 0.004) for HR with no interaction effect between *condition* and *time* (*p* = 0.287). The HR was significantly elevated compared to baseline across intensity conditions at the 1st and 2nd s post‐perturbation (*p* = 0.006 and *p* = 0.002, respectively). Also, the HR response was intensity‐dependent as HR was less elevated in the LOW intensity condition after the perturbation as compared to MED or HIGH (*p* = 0.035 and *p* = 0.012, respectively). There was no difference in how HR changed after the perturbation between MED and HIGH intensity conditions (*p* = 1.00).

**FIGURE 2:**
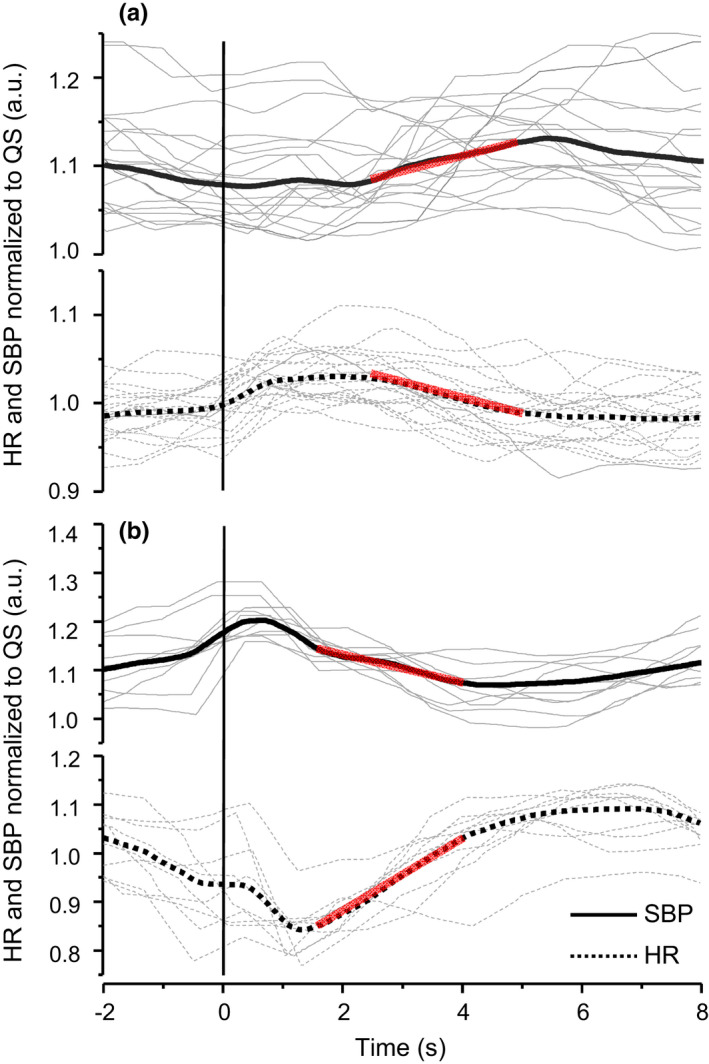
Examples of beat‐to‐beat SBP (solid line) and HR (dotted line) for 2 participants during MED perturbation demonstrating a commonly observed (a) and uncommonly observed (b) response. Data are presented as individual trials (grey lines) and the average (bolded lines). The vertical line represents the onset of the perturbation. The slopes (red lines) over the SBP and HR data show the sequences used to calculate cBRS

The inter‐individual variability in timing and direction of the cardiovascular response is illustrated in Figure 2. Two types of responses in SBP and HR to the perturbations were observed. A rapid tachycardia and delayed blood pressure response to postural perturbations occurred in most participants (Figure 2a), and a bradycardia response that aligned with a high SBP at the onset of the perturbation occurred in two participants (Figure 2b).

### 3.2 Perturbation effect on cardiac baroreflex response

The characteristics of the cardiac baroreflex sequence analysis are presented in Table 3. No effect of perturbation intensity was observed in cBRS gain (*p* = 0.570), the length of sequences (*p* = 0.723), the ratio between the number of sequences and the number of perturbation trials (*p* = 0.673), the percentage of up sequences (R‐R interval and SBP increased; *p* = 0.636), or the percentage of down sequences (R‐R interval and SBP decreased; *p* = 0.782). When the data were averaged across perturbation intensities, cBRS was elevated during perturbations compared to Baseline (*p* = 0.046) whereby, of the sequences identified in the post‐perturbation segment, the proportional number of up‐sequences was higher compared to Baseline (*p* < 0.0001). There was no difference in the length of sequences between perturbations and Baseline (*p* = 0.099; Table 4).

**TABLE 3:** The characteristics of cardiac baroreflex sensitivity measures from the sequence analysis during perturbation tests

	Perturbation intensity		
	LOW	MED	HIGH
Gain (ms/mmHg)	11.2 (6.1)	11.1 (6.5)	10.4 (4.9)
Length (# of RRIs)	3.6 (0.3)	3.6 (0.4)	3.6 (0.3)
Seq/Pert (%)	70 (27)	67 (23)	71 (20)
R^2^	0.94	0.95	0.95
Up Seq (%)	75 (15)	76 (20)	79 (19)
Down Seq (%)	27 (15)	27 (20)	24 (19)

Note

Data presented as mean (SD) for the LOW, MED, and HIGH. For all sequence characteristics *n* = 18, except for the Down Seq where *n* = 15.

Abbreviations: Down Seq, sequences where both RRI and Systolic Blood Pressure are decreasing expressed as the percentage of the total number of sequences; R^2^, goodness‐of‐fit measure for the linear regression models (range: 0–1);RRI, R‐R interval; Seq / Pert, ratio between the number of sequences to the number of perturbation trials; Up Seq, sequences where both RRI and Systolic Blood Pressure are increasing.

**TABLE 4:** The characteristics of cardiac baroreflex sensitivity measures from the sequence analysis during quiet standing and averaged across perturbation conditions

	Task (*n* = 19)		Quiet standing (*n* = 17)	
	Perturbation	Baseline	Baseline	Recovery
Gain (ms/mmHg)	10.6 (5.4)*	8.3 (2.4)	8.5 (3)	9.1 (3)
Length (# of RRIs)	3.6 (0.3)	3.8 (0.4)	3.7 (0.4)	3.6 (0.4)
Seq/Pert (%)	70 (20)	—	—	—
Total sequences (#)	53 (7)	75 (10)	75 (9)	71 (12)
R^2^	0.94	0.94	0.94	0.94
Up Seq (%)	77 (13)*	42 (5)	41 (4)	48 (4)*

Note

Data presented as mean (SD) for the averaged LOW, MED, and HIGH perturbations, Baseline, and Recovery conditions

Abbreviations: R^2^, goodness‐of‐fit measure for the linear regression models (range: 0–1);RRI, R‐R interval; Seq/Pert, ratio between the number of sequences to the number of perturbation trials; Up Seq, sequences where both RRI and Systolic Blood Pressure are increasing.

* Significant difference from Baseline (*p* < 0.05).

### 3.3 Stability of the baroreflex

Out of the 19 participants, 17 provided a complete data set for quiet standing (Table 4). All sequences during the 5‐min quiet standing conditions were analyzed. The time between Baseline and Recovery period recordings was a minimum of 15 min. There were no significant differences in cBRS (*p* = 0.216), sequence length (*p* = 0.810), and a total number of sequences (*p* = 0.243). However, during Recovery, the proportional number of up‐sequences were elevated (*p* = 0.008) compared to Baseline. The ICC for quiet stance cBRS between quiet standing conditions was excellent (ICC = 0.896; 95% CI = 0.715–0.962).

## 4 DISCUSSION

The primary results from the study indicated that, overall, postural perturbations induced transient but rapid HR acceleration, accompanied with a delayed SBP increase. Heart rate response was affected by perturbation intensity. Subsequently, HR was decreasing towards baseline when SBP was increasing. The resulting cBRS was unaffected by perturbation intensity but was elevated during perturbations compared to Baseline.

### 4.1 Perturbation intensity effect on cardiovascular response

To our knowledge, this is the first study to measure the cardiovascular response during periods of postural instability. The data indicate that postural perturbations exhibit a form of physical stress that is reactionary in nature, comparable to voluntary exercise (Morgan et al., 1973; Wong et al., 2007). Therefore, the mechanisms mediating these responses might be comparable.

The HR response to postural perturbations was intensity dependent, a pattern that was not observed in the SBP response. The initial cardiovascular response to postural perturbations encompassed rapid and immediate tachycardia post‐perturbation with the SBP elevation delayed by 2–4 s but both had recovered before the subsequent perturbation (Figure 1). A notable observation is the early and large rate of increase in HR, particularly the HIGH condition, that was recovering prior to, or consequent with, the rise in SBP. A second and unexpected observation was that of a “continuous” bradycardia during the immediate post‐perturbation stage if the perturbation was initiated concurrently with a spontaneous rise in SBP. These results will have affected the variability in SBP and HR responses to perturbations although they also indicate that the baroreflex was operating well in this phase.

The mechanisms mediating the early and late phases of cardiovascular response to the postural perturbations appear to be complex. First, the overall increase in cBRS suggests that the baroreflex was featured in the response. It might be argued that an expected outcome would be a reduction in cBRS in this period that scales with intensity, which is typical of exercise‐induced changes (Bringard et al., 2017). The resolution of this unexpected outcome might be found in the pattern of HR and SBP changes in this post‐perturbation period which suggest the potential for two mechanisms. First, the rapid HR response may be driven by the rapid vagal withdrawal common in volitional exercise, with slower adrenergic response in both cardiac and vascular smooth muscle (Borst & Karemaker, 1982; Faguis & Wallin, 1980; Qing et al., 2018; Stauss et al., 1997). Consequently, a rapid rise in HR (or reduction in R‐R interval) while SBP is relatively stable during the first 2–3 s of recovery would be quantified as a marked resetting of the baroreflex set point. The return of HR to baseline when SBP was rising would be predicted by a classic baroreflex mechanism that, likely, is returning to its baseline cBRS at this time.

Importantly, these studies were performed in the upright posture where a considerable reduction in vagal dominance over HR had already occurred (Zamir et al., 2017), but the speculation that the rapid HR response was related to vagal withdrawal is consistent with recent evidence that vagal input still exists during exercise stress such that baroreflex manipulations can be made across a range of elevations in HR (Raven et al., 2019). Second, the relatively concurrent reduction in R‐R interval and rise in SBP suggest baroreflex resetting is occurring (Raven et al., 2019). While the exact cause of baroreflex resetting is not fully understood, the invocation of a baroreflex resetting outcome immediately following the perturbation is consistent with the concept of a feed‐forward mechanism emanating from central neural sites (Krogh & Lindhard, 1913; Matsukawa, 2012; Migdal & Robinson, 2018). Whereas the muscle metaboreflex associated with fatiguing muscle contractions are also suspected in the baroreflex resetting process (Raven et al., 2019) it is unlikely that this mechanism participated in the current study because of the very brief and submaximal levels of the leg muscles during the approximately 8 s period of work. In contrast, the second phase of the post‐perturbation period is what would be expected from a baroreflex inhibitory effect whereby increasing SBP would result in bradycardia.

### 4.2 Perturbation effect on baroreflex response

The re‐establishment of HR and SBP after the initial response to the perturbation suggests the involvement of the cardiac baroreflex. As blood pressure increased, loading of the arterial baroreceptors would lead to deceleration of the heart. However, inter‐individual variability in the time course of HR and SBP indicated that the cardiac baroreflex can operate in response to elevated or depressed SBP post‐perturbation. The presence of baroreflex influence following a brief postural shift has been observed (Borst & Karemaker, 1982). The authors posited baroreflex‐mediated bradycardia occurred secondary to the initial pressor response in a sit‐to‐stand task. The elevated cBRS found in the current study indicates that the cardiac baroreflex can modulate greater changes in HR with similar changes to SBP. The benefit of elevated cBRS during postural perturbations might involve improved stabilization of arterial blood pressure.

The reasons for variability in cardiovascular response and timing of cardiac baroreflex involvement post‐perturbation remains speculative. The upright posture and nature of the study provides additional influences on cardiovascular control that must be considered. Ventilation is a known determinant of HR. Also, the sequence method is influenced by respiration (Silva et al., 2019), although, the similar length of sequences during the conditions makes it unlikely that there was a large respiration effect on the cardiac baroreflex. Contributions from the vestibular system and emotional arousal may have impacted the results, both of which provide neural inputs into the brainstem nuclei that form the neural pathway of baroreflex function (Benarroch, 2018; McCall et al., 2017; Yates, 1996). Although acute psychological stress decreases cBRS in healthy adults (Truijen et al., 2011; Virtanen et al., 2003), in balance studies, anxiety created by fear of falling was correlated with blood pressure rises that influenced the selection of balance strategy (Carpenter et al., 2006).

### 4.3 Stability of the baroreflex

The stability of the cardiac baroreflex measured during quiet standing before and after the perturbation conditions was strong, with an ICC value of 0.896. These data support the ability of the current protocol to evoke rapid and brief changes in cBRS. Also, the pre and post perturbation values of cBRS were similar to other studies that measured cBRS in upright standing (Bringard et al., 2017; Xu et al., 2017), although our values were on the lower end of the spectrum.

### 4.4 Limitations

The specific impact of respiration on the current data was not studied and breathing patterns were spontaneous. The perturbations were applied without reference to respiration, probably affecting the variability in R‐R interval and SBP data. While the specific effects of breathing on the current outcomes are not known, they are expected to be diminished through the averaging of multiple trials in each condition. Also, the cardiovascular responses appear to have been affected by the timing of the perturbations relative to spontaneous fluctuations in SBP. Lastly, it is not known if the cardiovascular response observed was due to postural shifts or a defence/alerting response. Additional studies are required to address these issues. The study results are delimited by the choice of perturbations and the state of health across the participants.

### 4.5 Perspectives

A novel approach to study cardiovascular response during active postural perturbations was conducted in healthy young adults. Results indicated central and reflex mediated hemodynamic response and recovery to postural perturbations. The importance of this regulatory mechanism in balance control may relate to the prevalence of falls in older adults who have been shown to demonstrate impaired cardiac baroreflex function in standing balance studies (Verma et al., 2017, 2019). Populations with balance deficits, such as older adults (Lord et al., 1991), are known to express an attenuated HR response (Muller et al., 2012) and altered blood pressure regulation (i.e., increased vascular resistance, and decreased ability for the heart to respond to acute fluctuations in blood pressure; Monahan, 2007; Rodrigues et al., 2018).

## 5 CONCLUSION

The results of this study provide evidence that HR response is intensity‐dependent, and that the cBRS is elevated compared to quiet standing but remains relatively constant across the three levels of perturbation used in the current study. Maintaining balance during unexpected postural perturbations increased HR, followed by an increase in SBP. The mechanism is unknown, but it is speculated that the initial cardiovascular response to perturbations was followed by a robust secondary cardiac baroreflex response that drove HR and SBP recovery. In addition, the stability of the cardiac baroreflex during quiet standing on a treadmill was strong. Therefore, monitoring cardiovascular response and cBRS during balance may be of interest and future studies can utilize this protocol's design.

## CONFLICT OF INTEREST

The authors do not have any conflicts of interest to disclose.

## AUTHOR CONTRIBUTIONS

Patrick Siedlecki conceived, designed, and conducted the experiment. Patrick Siedlecki and Tanya D. Ivanova analyzed data. Patrick Siedlecki, Tanya D. Ivanova, J. Kevin Shoemaker, and S. Jayne Garland interpreted the results of the experiment. Patrick Siedlecki wrote the drafted manuscript. S. Jayne Garland supervised the study. All authors revised, read, and approved the submitted version.
